# Acute Kidney Injury and Hepatorenal Syndrome in Patients with Cirrhosis

**DOI:** 10.3390/jcm13010199

**Published:** 2023-12-29

**Authors:** Nazli Begum Ozturk, Ece Janet Dinc, Abhishek Swami, Ahmet Gurakar

**Affiliations:** 1 Department of Internal Medicine, Beaumont Hospital, Royal Oak, MI 48073, USA; 2 School of Medicine, Baskent University, 06790 Ankara, Turkey; 3 Division of Nephrology, Beaumont Hospital, Royal Oak, MI 48073, USA; 4 Division of Gastroenterology and Hepatology, Johns Hopkins University Hospital, Baltimore, MD 21287, USA

**Keywords:** cirrhosis, hepatorenal syndrome, terlipressin, acute kidney injury, liver disease

## Abstract

Acute kidney injury (AKI) is common in hospitalized patients with cirrhosis. Hepatorenal syndrome (HRS) is a type of AKI known as HRS-AKI. It is a severe complication of cirrhosis with high morbidity and mortality. While certain vasoconstrictor medications have been shown to improve HRS-AKI, no clear transplant-free survival benefit has been reported with medical therapies. Patients with HRS-AKI should be considered for urgent liver transplantation evaluation. In this review, we discuss the most recent updates on the definition, diagnosis, and management of AKI in cirrhosis, with special a emphasis on HRS.

## 1. Introduction

Acute kidney injury (AKI) is common and can be seen in up to 50% of hospitalized patients with cirrhosis, and is associated with high morbidity and mortality (Table 1) [1,2,3,4]. AKI can be categorized into three main causes: renal hypoperfusion (prerenal AKI), often due to hypovolemia; intrinsic/structural kidney injury; and postrenal injury, due to mechanical obstruction. In patients with cirrhosis and AKI, hypoperfusion due to hypovolemia accounts for approximately 50% of the cases, intrinsic causes account for approximately 30%, hepatorenal syndrome (HRS) accounts for 15–20%, and post-renal injury accounts for <1% [1,5]. HRS is a unique cause of AKI in patients with cirrhosis resulting from hypoperfusion caused by renal vasoconstriction. HRS is classified into two categories: HRS-AKI and HRS-chronic kidney disease (HRS-CKD). HRS-AKI is more commonly seen after a decrease in effective arterial volume (e.g., diuretic-induced, gastrointestinal bleeding, excessive lactulose use, large-volume paracentesis), systemic vasodilation (sepsis, neurogenic shock), and medications causing intrarenal vasoconstriction (nonsteroidal anti-inflammatory drugs, renin–angiotensin–aldosterone system blockers); however, it can also be seen without obvious risk factors. Patients with ascites have an 8% annual risk for HRS, and 1.2% of hospitalized patients with cirrhosis have HRS [6]. The prognosis is poor after the diagnosis of HRS-AKI, with a median survival of two weeks after the diagnosis [6,7].

## 2. Assessment of Kidney Function in Patients with Cirrhosis

Assessment of kidney function in patients with cirrhosis is challenging. Serum creatinine (sCr) overestimates the glomerular filtration rate (GFR) in patients with cirrhosis due to multiple reasons [5,7,9,10]. First, sCr production from the liver is impaired in the setting of cirrhosis. Second, many patients with cirrhosis have sarcopenia with increased protein catabolism and protein-calorie malnutrition, which contributes to reduced levels of sCr. Third, the presence of elevated serum bilirubin can cause inaccurate measurement of sCr. In addition, an increase in creatine can lag in patients with fluid overload and AKI despite a true decrease in GFR. eGFR is calculated using creatine, cystatin C, or both and is based on stable levels in the general population. Cystatin C is a low molecular weight protein that is almost exclusively eliminated by glomerular filtration [11]. In patients with cirrhosis, the performance of eGFR based on cystatin C was lower compared to the general population [12]. Moreover, eGFR equations overestimate true GFR in patients with cirrhosis, in particular in patients with GFR < 40 mL/min/1.73 m^2^ or ascites, and should be used cautiously.

## 3. Definition and Diagnosis of Hepatorenal Syndrome

The definition and diagnosis of HRS have been revised in recent years by the International Club of Ascites (ICA) (Table 2) [8]. HRS is now classified into two groups: HRS-AKI (formerly known as HRS type 1 or acute HRS) and HRS-non-AKI (HRS-NAKI, formerly known as HRS type 2 or chronic HRS) (Table 3). American Association for the Study of Liver Diseases (AASLD) also defines HRS-AKI according to the ICA criteria: an increase in sCr ≥ 0.3 mg/dL within 48 h or an increase in sCr ≥ 1.5 times from baseline sCr that is known or presumed to have occurred within the preceding 7 days [3]. HRS-NAKI is then further divided into HRS-acute kidney disease (HRS-AKD), which is defined when eGFR < 60 mL/min per 1.73 m^2^ for <3 months, and HRS-CKD, which is defined when eGFR < 60 mL/min per 1.73 m^2^ for >3 months [1,2,8].

Formerly used definitions/diagnoses for HRS were made when a rapid reduction in kidney function with an increase in sCr > 2.5 mg/dL was present for <2 weeks for HRS-1 and more chronic deterioration in kidney function for HRS-2 [1]. The new definition of HRS eliminates the threshold for sCr for the diagnosis of HRS and allows earlier diagnosis for patients with normal sCr but reduced kidney function, such as women, older patients, and patients with sarcopenia [1]. In addition, a fixed sCr threshold in the former definition of HRS in the absence of dynamic changes does not differentiate acute vs. chronic kidney injury [8].

## 4. Pathophysiology of Hepatorenal Syndrome

**Figure 1:**
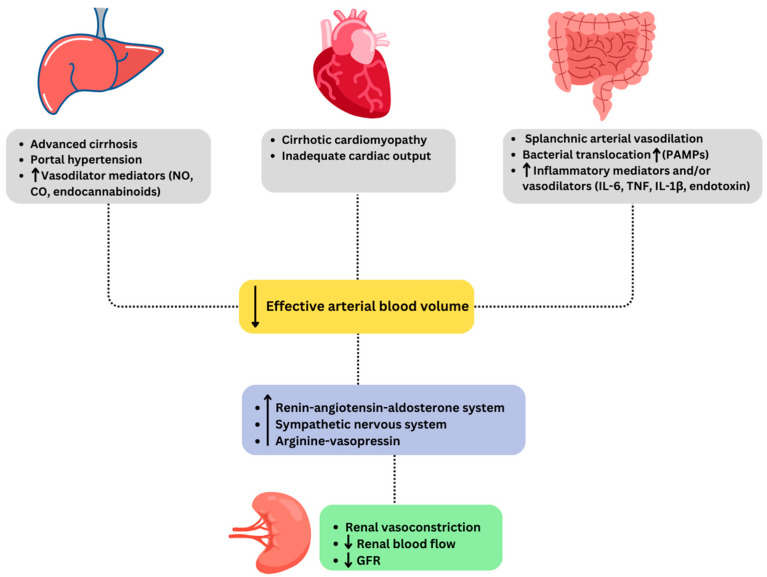
Pathophysiology of hepatorenal syndrome. Abbreviations: CO: carbon monoxide, GFR: glomerular filtration rate, IL: interleukin, NO: nitric oxide, PAMPs: pathogen-associated molecular patterns, TNF: tumor necrosis factor.

“Hepatorenal physiology” represents renal vasoconstriction in an attempt to increase renal perfusion due to systemic arterial vasodilation and cirrhosis-associated cardiomyopathy in patients with cirrhosis, summarized in Figure 1 [3]. In addition to circulatory dysfunction changes, a systemic inflammatory state has been shown to increase the susceptibility for HRS [14]. Patients with cirrhosis who have ascites, particularly those who have refractory ascites, have the highest risk of AKI, in particular HRS-AKI, due to hemodynamic alterations caused by portal hypertension. Cirrhosis causes increased intrahepatic resistance due to distortions of the liver architecture resulting from fibrosis and a paradoxical splanchnic and systemic vasodilation due to increased production of nitric oxide, prostacyclins, carbon monoxide, and endocannabinoids [1,10,15]. Compensatory hyperdynamic circulation initially preserves the effective intravascular volume in the early stages of cirrhosis. As cirrhosis progresses, hyperdynamic circulation cannot compensate for the splanchnic vasodilation and results in the activation of the renin–angiotensin–aldosterone system (RAAS), sympathetic nervous system (SNS), and at later stages, inducing non-osmotic secretion of arginine–vasopressin (AVP) to maintain increased effective arteriolar volume. These compensatory mechanisms cause vasoconstriction and sodium and water retention in an attempt to counteract vasodilation, resulting in refractory ascites. Moreover, these vasoconstrictive systems (mainly renin and angiotensin) cause renal vasoconstriction, decrease renal blood flow, and result in kidney injury. In addition, these mechanisms often cannot compensate to maintain perfusion and contribute to circulatory dysfunction and worsening renal function [2]. Another mechanism is the presence of cirrhotic cardiomyopathy, as patients with cirrhosis are in a state of hyperdynamic circulation with increased cardiac output and decreased peripheral resistance and mean arterial pressure [16]. Cirrhosis-induced cardiomyopathy can be seen in up to 50% of patients with cirrhosis [17]. As cardiac function deteriorates, the first manifestation of systolic dysfunction due to myocyte damage from sympathetic overactivity followed by diastolic dysfunction is seen. Infections such as spontaneous bacterial peritonitis (SBP) release tumor necrosis factor alpha (TNF) that can exacerbate cardiomyopathy. The decreased cardiac output is an important contributor to HRS as reduced stability of the circulatory system determines renal blood flow and function [2,18]. Moreover, although cardiac compensatory mechanisms may be able to maintain adequate perfusion in healthy individuals, they may not be efficient in patients with cirrhosis due to multiple stressors, including infections, in particular with SBP, gastrointestinal bleeding, or medications including beta blockers, diuretics, and angiotensin-converting enzyme inhibitors [17].

In patients with cirrhosis, neurohormonal systems are activated to counteract decreased effective arteriolar volume caused by peripheral vasodilation. The RAAS, SNS, and AVP play significant roles as part of these neurohormonal systems and lead to the progression of cirrhosis [19]. Initially, despite decreased renal blood flow, angiotensin II-mediated effector arteriole vasoconstriction and nitric oxide-mediated afferent arteriole vasodilation preserve GFR [20]. With the progression of cirrhosis, GFR progressively declines due to disruptions in nitric oxide production and gives rise to renal cortical ischemia, which is the hallmark of HRS. If the decrease in renal blood flow is not restored in a timely manner, continued vasoconstriction and resulting renal ischemia can also lead to ATN, which is the most common type of intrinsic AKI.

## 5. Diagnostic Workup of Acute Kidney Injury in Patients with Cirrhosis

HRS-AKI is diagnosed in the setting of cirrhosis and ascites, and the diagnosis of AKI is based on the ICA-AKI criteria [8]. In contrast to The Kidney Disease: Improving Global Outcomes criteria for AKI, ICA criteria do not consider decreased urinary output as part of the diagnostic criteria for AKI in patients with cirrhosis, as patients with cirrhosis are expected to have lower urine output at baseline due to avid sodium and water retention, and most patients being on diuretics [8,10,21]. The absence of shock, no current or recent nephrotoxic drugs use, absence of proteinuria (>500 mg/day), absence of microhematuria (>50 RBCs/per high-power field), absence of active sediments in urine, absence of white blood cell casts in the urine, and normal renal imaging should be present [8].

### Conventional and Emerging Biomarkers

Conventional tests such as sCr, urine output, fractional excretion of sodium (FeNa), fractional excretion of urea (FeUrea), and proteinuria >500 mg to define the phenotypes of AKI have limited value in patients with cirrhosis compared to the general population [11]. In general, FeNa is used to help differentiate prerenal and intrinsic AKI. FeNa < 1% suggests prerenal AKI, while >1% suggests intrinsic AKI in patients without liver disease [5]. However, almost all patients with advanced cirrhosis, particularly those with ascites, already have avid sodium retention and have FeNa < 1%, even in the absence of AKI or in the setting of ATN [1,5]. In addition, FeNa may be artificially elevated due to diuretics [11].

Differentiating HRS-AKI vs. ATN can be challenging, however FeNa < 0.1%, fractional excretion of urea < 21%, and fractional excretion of albumin < 44 mg/dL may aid in identifying HRS-AKI [22]. It should be noted that these cut-off values do not have high sensitivity or specificity in all cases. All attempts should be made to exclude prerenal AKI by a trial of volume expansion and stopping diuretics. In addition, glomerular etiology should be excluded by the absence of hematuria and proteinuria [5]. Decreased serum protein concentration is commonly seen in cirrhosis due to impaired liver synthetic function and/or malnutrition, and the cut-off value for non-physiologic proteinuria in patients with cirrhosis is not determined [11]. If urine examination raises a high suspicion of a glomerular disease, a renal biopsy is often needed to establish an accurate diagnosis.

In addition to standard blood and urine tests, several biomarkers, including urinary neutrophil gelatinase-associated lipocalin (NGAL), interleukin-18 (IL-18), kidney injury molecule 1 (KIM-1), liver-type fatty acid binding protein (L-FABP), trefoil factor 3, and glutathione-S-transferase-π have been shown to be elevated in ATN and HRS-AKI [23].

NGAL is the most extensively studied and promising biomarker of tubular injury in AKI [11]. In animal and human studies, urinary concentrations of NGAL were observed to be markedly increased (within 2 h) following ischemia [11]. In addition, human studies reported serum or urinary NGAL to be useful for detecting AKI at early stages in multiple clinical conditions, including sepsis, septic shock, and cardiac surgery, and also to identify AKI early in patients with liver cirrhosis and to differentiate ATN from HRS [11,24,25]. Urinary NGAL has been shown to be higher in patients with cirrhosis and AKI compared to cirrhosis without AKI, and within those with AKI, it was found to be markedly higher in patients with ATN compared to HRS, prerenal AKI or CKD [26,27]. However, urinary NGAL can be elevated in acute and chronic inflammation and also in the setting of CKD [28]. Moreover, although urinary NGAL is markedly elevated in ATN, there is a significant overlap when compared to HRS and other etiologies of ATN, particularly with the serum NGAL levels [11,27]. In addition, liver synthesis of NGAL increases if sepsis is present [29].

IL-18 is a proinflammatory cytokine that is overexpressed in the proximal tubule and is released into urine during AKI [30]. In patients with cirrhosis, higher urinary IL-18 levels have been shown in patients with ATN compared to other causes of AKI [31]. However, like urine NGAL, there is an overlap between ATN and non-ATN AKI for IL-18.

KIM-1 is a transmembrane protein that is upregulated during ischemic kidney injury and is a marker for proximal tubular injury [11]. Urinary KIM-1 has been shown to be elevated in patients with ATN compared to no increase in patients with prerenal AKI or CKD. However, in patients with cirrhosis, levels were increased in ATN compared to other causes of AKI [11]. Similar to NGAL and IL-18, significant overlap exists for KIM-1 for patients with ATN compared to non-ATN-AKI.

These tests may help identify AKI earlier compared to sCr and are promising; however, they are not widely available, and none of them are specific [3,11,32,33]. The majority of the studies comparing ATN vs. other etiologies lack histological confirmation of ATN. In addition, with the majority of biomarkers, there remains a substantial overlap between the groups, which is a limitation for the tests.

## 6. Acute Kidney Injury in Patients with Acute-on-Chronic Liver Failure

AKI is common in patients with acute-on-chronic liver failure (ACLF), and it is reported to be present in 69% of patients with ACLF, according to the CANONIC study, and is one of the defining features of ACLF [34]. In addition, the degree of AKI and renal function are the most important factors in the prognosis of ACLF [23,35]. Uncontrolled inflammation worsens the hemodynamics and aggravates kidney injury in patients with ACLF. A profound systemic inflammatory state with significantly elevated levels of cytokines is more commonly seen in ACLF compared to non-ALCF hepatic decompensation. Patients with ACLF have higher levels of systemic inflammation, and the presence of renal dysfunction was reported to be correlated with IL-6, IL-8, and human nonmercaptalbumin [2,36]. Moreover, elevated bile acid levels, worsening portal hypertension, cardiac dysfunction and resulting renal hypoperfusion, requirement for vasopressors, and extrarenal organ failure in ACLF can worsen renal dysfunction [23].

## 7. Prevention of Acute Kidney Injury and Hepatorenal Syndrome-Acute Kidney Injury

To prevent kidney dysfunction in patients with cirrhosis, precipitating factors for AKI and HRS-AKI, including gastrointestinal bleeding, bacterial infections, and large-volume paracentesis without adequate albumin administration, should be avoided [3]. Avoidance of nephrotoxic medications and maintaining appropriate volume status are important. The nephrotoxicity of contrast agents is controversial in patients with cirrhosis [37]. The presence of tense ascites may increase intra-abdominal pressure, lead to increased renal venous pressure, and may lead to AKI [38]. Large-volume paracentesis (>5 L) can increase vasodilatory state and can lead to worsening kidney function. Intravenous albumin 25% (4–6 g per L beyond 5 L of ascites removed) should be administered to prevent post-paracentesis circulatory dysfunction and hypovolemia. Albumin along with antibiotics should be administered in patients with SBP (1.5 g/kg of body weight daily on day one and 1 g/kg of body weight daily on day three) to reduce the incidence of renal impairment and mortality [39]. It should be noted that administration of albumin in patients with bacterial infections other than SBP has not been shown to prevent SBP or improve survival in patients with cirrhosis [3,40,41].

## 8. Management of Acute Kidney Injury and Hepatorenal Syndrome-Acute Kidney Injury

Once AKI is diagnosed, medications that can precipitate or worsen AKI should be discontinued. As HRS is a diagnosis of exclusion, HRS-AKI should be treated when other causes of AKI have been excluded. Diuretics, nonselective beta blockers, vasodilators, and nonsteroidal anti-inflammatory drugs (NSAIDS) should be promptly discontinued. Aminoglycosides, vancomycin, and amphotericin B have direct renal tubule toxicity, and beta-lactam antibiotics, proton pump inhibitors, ciprofloxacin, NSAIDs, and acyclovir can cause acute interstitial nephritis and should be used with caution [5]. If patients have other concomitant organ failures, management in an intensive care unit can be considered. If hypovolemia persists, lactulose can be held, or a dose reduction can be considered. In addition, infections, in particular, SBP, should be ruled out, and all patients with cirrhosis and ascites should undergo a diagnostic paracentesis on admission as most patients with cirrhosis do not have fever or abdominal pain to point toward SBP. Other infections should also be excluded, and a chest X-ray with respiratory, blood, and urine cultures should be collected. Empirical antibiotics should be started if there is a high suspicion of infection.

Volume expansion is essential in patients with AKI due to hypovolemia, and response to volume expansion helps determine the cause of AKI. As prerenal AKI due to hypovolemia is the most common cause of AKI in patients with cirrhosis, a volume expansion for 24 or 48 h is recommended in patients who are hypo- or euvolemic. The type of fluids (crystalloids vs. albumin) and the rate should be individualized according to the patient’s volume status and cause of AKI. Intravenous albumin at the dose of 1 g/kg of body weight daily (maximum 100 g) should be given for two consecutive days for volume expansion in most patients. Volume status should be optimized to prevent worsening renal function and to avoid fluid overload, worsening ascites, heart failure, and pulmonary edema [5]. However, assessing volume status in patients with cirrhosis is also challenging as there is no validated monitoring tool in this patient population [5]. Urine output should be monitored as oliguria is associated with poor prognosis [42].

HRS is potentially reversible by vasoconstrictor medications and liver transplantation (LT). Patients with stage 2 or 3 AKI meeting the criteria for HRS-AKI are candidates for vasoconstrictive therapy [43]. Both AASLD and the European Association for the Study of Liver guidelines recommend terlipressin plus albumin as the first-line treatment for HRS, and LT is the best therapeutic option for patients with HRS, independent of the response to the medical therapy [3,37]. The mainstay of the treatment of HRS-AKI is to improve renal blood flow by increasing effective circulation volume through the use of albumin and systemic vasoconstrictors to counteract arterial and splanchnic vasodilation [10,23]. Multiple studies noted that the degree of improvement in mean arterial pressure is correlated with improvement in renal function [44]. However, it should be noted that the use of vasoconstrictors has not been shown to improve long-term survival and should not be considered as a cure but rather as a bridge to transplantation [10].

### 8.1. Terlipressin

Terlipressin, a vasopressin 1A receptor analog on vascular smooth muscle cells, is considered a first-line agent for HRS-AKI and primarily acts as a splanchnic vasoconstrictor and decreases portal and intrahepatic pressures. Terlipressin has been available in Europe for more than 20 years and used for the treatment of HRS; however, it was approved in the United States of America (USA) by the Food and Drug Administration for the treatment of HRS in September 2022. Terlipressin can be administered as a continuous infusion or as a bolus with similar efficacy; however, adverse effects have been shown to be lower with continuous infusion due to lower doses needed [45]. In addition, continuous infusions have been reported to lead to sustained suppression of portal pressure with a lower dose compared to bolus therapy [46]. In the USA, terlipressin is only approved for bolus administrations [43]. It can be administered through a peripheral intravenous line and does not require continuous cardiac monitoring. Terlipressin can be started as an initial bolus with 0.1–1 mg every 4–6 h [5]. According to the response to treatment, the initial dose can be increased, maintained, or discontinued. After three days of treatment, if sCr has decreased by 25% or more, the treatment dosing should be maintained until sCr < 1.5 mg/dL or up to 14 days if partial or non-response [5]. Partial response is defined as >50% sCr improvement without sCr < 1.5 mg/dL. If sCr has not improved by 25% by day four, the terlipressin dose can be increased by 2 mg every 6 h. If there is no improvement in sCr by day four, terlipressin should be permanently discontinued [43,47].

CONFIRM (a multicenter, randomized, placebo-controlled, double-blind study to confirm efficacy and safety of terlipressin in subjects with hepatorenal syndrome type 1) trial, the largest North American trial of terlipressin, involving 300 patients with cirrhosis and HRS-1, compared terlipressin and placebo reported that terlipressin improved kidney recovery in 30% vs. 18% compared to placebo, however it was also associated with increased death due to respiratory failure, 11% vs. 2% compared to placebo, mainly in patients with grade 3 ACLF [48]. One of the drawbacks was that the trial used sCr > 2.25 mg/dL to define HRS-1. Another RCT conducted using the HRS-AKI diagnostic criteria compared terlipressin and norepinephrine in patients with ACLF and HRS-AKI reported that terlipressin was associated with improved HRS reversal, need for RRT, and improved 28-day survival [49]. Reversal rates for HRS-1 were reported to be 36–55% in previously published RCTs comparing terlipressin and placebo with albumin [50].

Albumin should be concurrently administered with terlipressin at a dose of 20–40 g/daily. The role of albumin, in addition to its oncotic property resulting in volume expansion, is also in its non-oncotic properties such as antioxidant, immunomodulating and endothelium protective functions [51]. Long-term use of albumin in both inpatient and outpatient settings is not recommended. The ATTIRE (Albumin to Prevent Infection in Chronic Liver Failure) trial involving IV albumin administration in hospitalized patients targeting albumin >3 g/dL showed that it did not improve outcomes; however, it increased the risks of AKI and pulmonary edema [52].

Administration of terlipressin and albumin is more effective in HRS than terlipressin alone [53]. High rates of response to terlipressin have been reported in patients with sCr < 5 mg/dL, baseline bilirubin < 10 mg/dL, lower stage of ACLF, and sustained increase in mean arterial pressure (MAP) by 5–10 mm Hg with treatment [47,54,55]. Terlipressin is not recommended in patients with sCR > 5 mg/dL as it is reported to be ineffective [48]. Discontinuation of terlipressin is needed in 4–22% of patients [10]. As terlipressin is a vasoconstrictor, it can cause coronary, splanchnic, or peripheral extremity ischemia and is contraindicated in patients with a history of coronary, mesenteric, and peripheral ischemia [43]. Terlipressin can also lead to an increased incidence of respiratory failure due to pulmonary edema. Daily volume status should be carefully assessed (hypoxia, jugular venous distention, pulmonary vascular congestion on chest radiography, elevated right ventricular systolic pressure on echocardiography), especially in the setting of concurrent albumin administration. SpO_2_ should be monitored, and terlipressin should be discontinued if SpO_2_ < 90% [43,56].

### 8.2. Norepinephrine

Norepinephrine is preferred when terlipressin is not available. Norepinephrine can be used as a continuous intravenous infusion and is usually used in an intensive care setting with the goal of achieving an increase in MAP of 10 mm Hg or higher or an increase in urine output of >200 mL in 4 h [3]. Data regarding the effectiveness of norepinephrine compared to terlipressin are scarce; however, they appear to be equally effective in two controlled clinical trials [57,58]. However, norepinephrine requires continuous infusion in an intensive care unit setting and requires central venous access. An RCT comparing terlipressin vs. norepinephrine in 46 patients with HRS-1 reported that the rate of achieving Cr < 1.5 mg/dL was 39.1% vs. 43.4% [57]. In a meta-analysis involving 778 patients, norepinephrine 0.5–0.3 mg/h was non-inferior to terlipressin [59]. Norepinephrine can cause tachyarrhythmia, with 10% of the patients requiring discontinuation in studies [60].

### 8.3. Midodrine and Octreotide

If neither terlipressin nor norepinephrine is available, a trial of midodrine and octreotide can be used. Midodrine, an a1-agonist, is an orally available vasoconstrictor and can be used in combination with octreotide, a somatostatin analog, subcutaneously or intravenously for the treatment of HRS. Midodrine can be given 5 to 15 mg every 8 h with octreotide (100 to 200 μg every 8 h subcutaneously or 50 μg every hour intravenously). The combination of midodrine and octreotide has a weak vasoconstrictive effect, and multiple reports and meta-analyses of RCTs showed inferiority to terlipressin in the reversal of HRS-AKI [61]. As a result, octreotide and midodrine should only be used when terlipressin is unavailable.

## 9. Transjugular Intrahepatic Portosystemic Shunt

Transjugular intrahepatic portosystemic shunt (TIPS) may improve kidney function by reducing portal pressure, redistributing blood volume, and improving cardiac output; however, evidence is not sufficient to recommend TIPS for HRS-AKI, as data include a small number of patients, and studies are uncontrolled [62]. A meta-analysis involving 128 patients treated with TIPS for HRS-AKI reported that TIPS can be effective in reversing HRS-AKI with improvement in 93% of the patients; however, there was no control group, the patient population was heterogenous, and a high mortality rate was observed [5]. In addition, TIPS insertion was associated with significant risks in patients with AKI, including 90-day mortality of 25–80%; however, it is difficult to attribute what degree of high mortality was a result of the TIPS procedure [63].

## 10. Renal Replacement Therapy

Renal replacement therapy (RRT) is indicated in patients with HRS-AKI who are non-responsive to medical therapy and those with refractory volume overload, uremic symptoms, diuretic intolerance, electrolyte derangements including severe acidosis, hyponatremia, hyperkalemia that are not improving despite medical management [3,64]. There is no consensus on when to initiate RRT in patients with cirrhosis and AKI or HRS-AKI, and the optimal timing of RRT has not been studied in patients with cirrhosis [3]. RRT has typically been considered a bridge to LT, and its utility is controversial in patients who are not LT candidates due to risks, complications, and mortality rates in critically ill patients [64,65]. RRT does not improve survival in patients with HRS-AKI if medical therapy fails [64]. A consensus report from the Acute Dialysis Quality Initiative group recommends withholding RRT unless there is an acute reversible component or plan for LT, as there is a lack of evidence for a survival benefit of RRT in HRS-AKI [66].

## 11. Liver Transplantation

LT is the treatment of choice and is the definitive treatment for patients with HRS, as it theoretically restores hepatorenal physiology. However, up to 25% of patients with HRS continue to have renal dysfunction after LT [67]. Kidney recovery after LT is not always predictable and is dependent on multiple clinical factors, such as the presence of CKD, diabetes, intrinsic renal injuries, procedure-related events, and post-LT immunosuppression [3]. The strongest predictor of no kidney recovery is the duration of pre-LT RRT, with each additional day of pre-LT RRT increasing the risk of no kidney recovery by 6% [68]. Pre-LT kidney function is a predictor of outcomes, shorter duration of HRS (<4 weeks) with better outcomes compared to >6 weeks of sustained AKI [41,67]. Simultaneous liver–kidney transplantation (SLK) can be considered for patients with prolonged renal dysfunction before LT, as the longer duration of HRS-AKI and higher pre-transplant sCr have been shown to be associated with worse outcomes [67]. SLK may improve post-LT outcomes in patients with liver cirrhosis and concomitant kidney dysfunction. In the USA, the Organ Procurement Transplant Network implemented a new policy for SLK for transplant centers to use as a unified criteria for the selection of patients for SLK in 2017. This policy also implemented a safety net to allow time for kidney recovery post-LT and, in the event of persistent kidney dysfunction post-LT, to prioritize these patients for kidney transplantation.

## 12. Future Directions

Given the recent changes in the definition and diagnosis of HRS-AKI no longer require a set sCr over 1.5 mg/dL threshold, the goal is to identify HRS-AKI early and initiate treatment to increase the chances of HRS-AKI reversal. Although ICA guidelines provide the framework for the diagnosis of HRS, several limitations still exist. As HRS is considered a diagnosis of exclusion, and a 48 h empirical fluid challenge is required for the diagnosis, this approach may delay the initiation of vasoconstrictor in patients with HRS or worsen the volume overload in patients who are unlikely to respond, such as ATN [69]. There is a need for a reliable biomarker to distinguish HRS from ATN. Tubular injury markers such as NGAL are not approved for clinical use worldwide. As none of the biomarkers for phenotyping AKI in cirrhosis are specific for the etiology of AKI, a combination of urinary and/or serum biomarkers may be more accurate than a single biomarker. Incorporating emerging biomarkers and genomic analysis could also improve diagnostic accuracy.

Pathophysiology of HRS is complex and still not completely understood, with contributions from splanchnic vasodilation and decreased effective arterial blood volume and subsequent activation of vasoconstrictor systems causing systemic circulatory dysfunction; systemic inflammation, bacterial translocation, renal microcirculatory dysfunction, renal inflammation, and cardiac dysfunction due to cirrhotic cardiomyopathy [70]. Although splanchnic vasodilation and the resulting decrease in effective arterial blood volume and subsequent activation of RAAS, SNS, and circulatory dysfunction was believed to be the main issue for HRS, up to 64% of the patients do not have symptoms of improvement with volume expansion and treatment with splanchnic vasoconstrictors [65,70]. Identification of the mechanisms of these pathophysiologies may reveal new potential therapeutic targets for this complex condition.

High-quality randomized controlled trials for the management of HRS-AKI are lacking, and many recommendations are based on observational data or small trials, including heterogeneous patient populations from tertiary academic centers, along with expert opinions. The optimal dose, use of combination medications, monitoring, and assessing the adequate response to medical management still remains unknown. Head-to-head randomized controlled trials in patients with well-defined HRS-AKI could unravel and refine the optimal management. The predictors of response to the medical therapy are not standardized or robust, and lack validation. Development and validation of clinical scoring systems could help determine who will respond best to treatment. In addition, adverse effects of the current therapies are underreported, and more rigorous monitoring and standardized reporting are essential. Multicenter, collaborative studies remain important to achieve a large patient volume and can achieve more generalizable and broader evidence.

Post-LT recovery of kidney function remains challenging to predict before the transplant period, and consideration of SLK for those with persistent kidney dysfunction is critical. The optimal timing for LT in patients with HRS-AKI is unclear. Models incorporating transplant waitlist times and treatment response rates could provide more precise recommendations.

## 13. Conclusions

AKI is a common complication and is associated with poor survival in patients with cirrhosis. Patients with renal impairment with cirrhosis should be considered for LT. Despite fulfilling all the criteria for HRS, patients can still have structural kidney damage. The inclusion of dynamic sCr changes allows earlier diagnosis and earlier initiation of treatment with vasoconstrictor medications, which is the mainstay of treatment for HRS. Terlipressin should be administered as the first-line when available for the treatment of HRS, and norepinephrine should be administered if the patient is being treated in an intensive care unit, given the need for central access. Midodrine plus octreotide can be considered if terlipressin is not available and if the patient is not in an intensive care unit. Caution is needed for patients at risk for fluid overload, specifically with terlipressin. No vasoconstrictor therapy improves LT-free survival. RRT may be used as a bridge to LT; however, patients should not be maintained on long-term RRT as it has a negative effect on post-LT outcomes [65]. SLK should be considered in patients with prolonged pre-transplant RRT or CKD.

## Figures and Tables

**Table 1:** International Club of Ascites new definitions for the diagnosis and management of AKI in patients with cirrhosis.

Subject	Definition		
Baseline sCr	A value of sCr obtained in the previous three months, when available, can be used as baseline sCr. In patients with more than one value within the previous three months, the value closest to the admission time to the hospital should be used. In patients without a previous sCr value, the sCr on admission should be used as baseline.		
Definition of AKI	- Increase in sCr ≥ 0.3 mg/dL (26.5 μmol/L) within 48 h; or, - A percentage increase sCr ≥ 50% from baseline which is known, or presumed, to have occurred within the prior seven days		
Staging of AKI	- Stage 1: increase in sCr ≥ 0.3 mg/dl (26.5 μmol/L) or an increase in sCr ≥ 1.5-fold to 2-fold from baseline; - Stage 2: increase in sCr > 2-fold to 3-fold from baseline; - Stage 3: increase in sCr > 3-fold from baseline or sCr ≥ 4.0 mg/dL (353.6 μmol/L) with an acute increase ≥ 0.3 mg/dL (26.5 μmol/L) or initiation of renal replacement therapy		
Progression of AKI	Progression	Regression	
	Progression of AKI to a higher stage and/or need for RRT	Regression of AKI to a lower stage	
Response to treatment	No response	Partial response	Full response
	No regression of AKI	Regression of AKI stage with a reduction in sCr ≥ 0.3 mg/dL (26.5 μmol/L) above the baseline value	Return of sCr to a value within 0.3 mg/dL (26.5 μmol/L) of the baseline value

Abbreviations: AKI, acute kidney injury; CKD, chronic kidney disease; HRS, hepatorenal syndrome; RRT, renal replacement therapy; sCr, serum creatinine. (Adapted from Angeli et al. with permission from Elsevier [8]).

**Table 2:** Diagnostic criteria for HRS-AKI in patients with cirrhosis.

Diagnosis of cirrhosis and ascites
Diagnosis of AKI according to ICA-AKI criteria
No response after 2 consecutive days of diuretic withdrawal and plasma volume expansion with albumin 1 g per kg of body weight
Absence of shock
No current or recent use of nephrotoxic drugs (NSAIDs, aminoglycosides, iodinated contrast media, etc.)
No macroscopic signs of structural kidney injury ^a^ defined as: - Absence of proteinuria (>500 mg/day) - Absence of microhematuria (>50 RBCs per high-power field), - Normal findings on renal ultrasonography

Abbreviations: AKI, acute kidney injury; HRS, hepatorenal syndrome; ICA, International Club of Ascites; NSAID, nonsteroidal anti-inflammatory drug; RBC; red blood cell; sCr, serum creatinine. ^a^ Patients who fulfill these criteria may still have structural damage, such as tubular damage. Urine biomarkers will become an important element in making a more accurate differential diagnosis between HRS and acute tubular necrosis. (Adapted from Angeli et al. with permission from Elsevier [8]).

**Table 3:** New classification of HRS subtypes.

Old Terminology	New Terminology	Definition
HRS-1 ^a^	HRS-AKI	(a) Absolute increase in sCr ≥ 0.3 mg/dL within 48 h and/or (b) Urinary output ≤ 0.5 mL/kg B.W. ≥6 h ^b^ or (c) Percent increase in sCr ≥ 50% using the last available value of outpatient sCr within 3 months as the baseline value
HRS-2 ^a^	HRS-AKD	(a) eGFR < 60 mL/min per 1.73 m^2^ for <3 months in the absence of other (structural) causes
	HRS-NAKI	(b) Percent increase in sCr < 50% using the last available value of outpatient sCr within 3 months as the baseline value
	HRS-CKD	(a) eGFR < 60 mL/min per 1.73 m^2^ for ≥3 months in the absence of other (structural) causes

Abbreviations: AKD, acute kidney disease; AKI, acute kidney injury; CKD, chronic kidney disease; eGFR, estimated glomerular filtration rate; HRS, hepatorenal syndrome; NAKI, nonacute kidney injury; sCr, serum creatinine. ^a^ Fulfillment of all the new International Ascites Club criteria for the diagnosis of HRS. ^b^ The evaluation of this parameter requires a urinary catheter. (Adapted from Angeli et al. with permission from Elsevier [13]).

## Data Availability

Data are contained within the article.
